# High/low temperature setups for submillimetric samples under various extreme conditions at the AILES beamline

**DOI:** 10.1107/S1600577526004832

**Published:** 2026-06-05

**Authors:** Cecilia Taverna, Kelly Rader, Jean Blaise Brubach, Alexandre Voute, Mariia Dronova, Laurent Couillard-Labonnote, Quentin Libois, Pascale Roy, Marine Verseils

**Affiliations:** a Synchrotron SOLEIL 91190Saint-Aubin France; b Laboratoire d’Optique Atmosphérique 59655Villeneuve d’Ascq Cedex France; c Centre National de Recherches Météorologiques 31057Toulouse Cedex 1 France; University College London, United Kingdom

**Keywords:** high/low temperature, submillimetric samples, IR spectroscopy, THz spectroscopy

## Abstract

Infrared and terahertz spectroscopy performed at synchrotron facilities offers unique opportunities for probing matter under extreme and well controlled conditions. At the AILES beamline of Synchrotron SOLEIL, two complementary experimental setups have recently been developed to enable spectroscopy of submillimetric samples across a broad range of temperatures and pressures. These platforms operate from 10 K to 600 K and from vacuum up to 100 GPa, while accommodating a variety of sample environments, including liquid cells, diamond anvil cells and uniaxial strain devices. Combined with the high brilliance and stability of synchrotron radiation and advanced detection schemes, these setups are particularly suited for *in situ* and *operando* investigations, allowing real-time monitoring of structural changes, phase transitions and reaction dynamics under external stimuli. Their performance is illustrated through far-infrared measurements of water and ice over different thermodynamic states, demonstrating sensitivity to structural reorganizations and hydrogen-bond dynamics. These developments significantly expand the experimental capabilities of the AILES beamline and provide versatile tools for a wide user community, opening new perspectives for studies of functional materials, molecular systems and emerging phenomena under extreme conditions.

## Abstract 2

Two complementary high- and low-temperature experimental setups have been developed at the AILES beamline of Synchrotron SOLEIL to enable infrared and terahertz spectroscopy of submillimetric samples under extreme and well controlled pressure and temperature conditions (from 10 K to 600 K and from vacuum up to 100 GPa). The ensembles are compatible with a variety of sample environments, as liquid cells, diamond anvil cells and uniaxial strain devices.

## 1. Introduction

Infrared spectroscopy, particularly when conducted on a synchrotron beamline dedicated to the THz (25–70 cm^−1^), far- (70–500 cm^−1^) and mid-infrared (500–5000 cm^−1^) regions, offers remarkable experimental versatility. This technique is well suited for studying a wide range of systems, from functional materials to complex molecular assemblies. The main figures of merit are the flux and the brilliance of the source. Flux at the extraction was verified to be between ∼10^13^ to 10^14^ photons s^−1^ (0.1% bandwidth)^−1^. Concerning the brilliance, it was grossly evaluated to evolve from 10^19^ photons s^−1^ (0.1% bandwidth)^−1^ mm^−2^ steradian^−1^ for the near infrared (1 µm) to 10^13^ photons s^−1^ (0.1% bandwidth)^−1^ mm^−2^ steradian^−1^ for the far infrared. This is described in detail by Roy *et al.* (2006 ▸).

The exceptional brilliance of synchrotron radiation enables high spectral resolution experiments or investigations under extreme conditions (*e.g.* high pressure, low temperature, controlled atmospheres). These capabilities allow for the establishment of environments relevant to applications in solid-state physics, catalysis, materials science and astrophysics. Furthermore, the combination of *in situ* and *operando* techniques, coupled with advanced detection systems (such as bolometric or photoconductive detectors), facilitates real-time investigations. This includes tracking chemical reactions or phase transitions under external stimuli, such as pressure, temperature or light irradiation. Given the broad diversity of measurements performed on synchrotron beamlines, the AILES beamline of Synchrotron SOLEIL has recently developed two specialized setups. These experimental setups enable the investigation of samples in various physical states, accommodating submillimeter beam sizes while operating across an extensive range of conditions spanning temperatures from 10 K to 600 K and pressures from vacuum to 100 GPa.

This versatility facilitates precise control over experimental parameters, making them ideal for studying complex phenomena under extreme or finely tuned environments.

**Figure 1:**
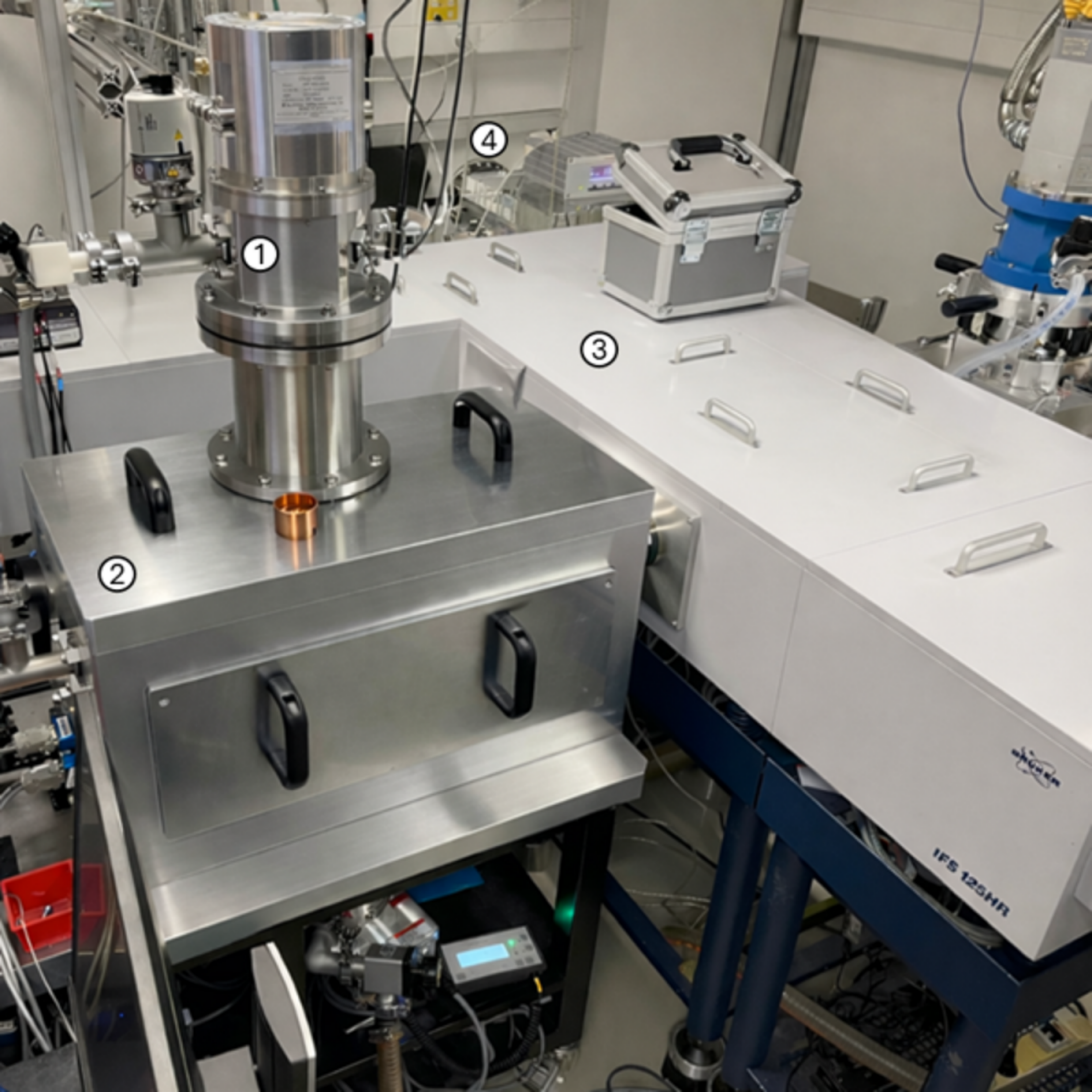
Photograph of the experimental station in low-temperature configuration: (1) cryostat, (2) optical setup box, (3) Fourier transform interferometer and (4) synchrotron beam entrance point.

At the AILES beamline, three interferometers (Bruker IFS125HR) are available: one for high-resolution gas-phase spectroscopy (maximum resolution 0.001 cm^−1^), enabling precise determination of molecular parameters relevant to atmospheric and astrophysical studies (Brubach *et al.*, 2010 ▸); the second interferometer is dedicated to *in situ* and *operando* experiments in the field of chemistry, and the last one is optimized for condensed-phase and materials spectroscopy, optimized for measurements under controlled temperature, pressure and humidity (Fig. 1 ▸). The beamline’s optical design and long-path interferometers permit high-flux operation across the THz to mid-IR range, complemented by multiple detectors (bolometric or photoconductive).

The beamline has developed specialized experimental stations optimized for submillimetric samples under well controlled extreme conditions of temperature and pressure (Fig. 1 ▸). These setups employ Schwarzschild optics, in a Cassegrain-type system, to focus synchrotron radiation onto samples as small as a few tens of micrometres, enabling high signal intensity even for very limited sample volumes. They support IR/THz spectroscopy in either transmission or reflectivity modes. The first version of this setup, mostly limited to high pressure experiments at low temperature, was described in a previous paper (Voute *et al.*, 2016 ▸). At present, two new ensembles, coupled with new versatile sample environments, are made available on the beamline as described here.

One setup is equipped with a pulse-tube cryostat allowing temperature measurements between 10 K and 300 K, while the other, coupled with a heating system, enables higher-temperature measurements from room temperature up to 600 K. Both setups are compatible with a variety of custom sample supports and can accommodate solid or liquid samples. The supports can also accept diamond anvil cells (DACs) for pressure measurements or a uniaxial cell for strain studies.

Both configurations enable *in situ* regulation of the sample’s temperature and pressure, thereby ensuring precise and reliable characterization under the targeted experimental conditions. These versatile capabilities open new opportunities to study phenomena that are highly sensitive to external conditions. In the following, we present an overview of the new experimental setups at the AILES beamline, describing their optical configurations and versatile sample environments, including liquid cells for liquid and thin-film studies, DACs for high-pressure measurements, and uniaxial strain cells for strain deformation experiments. We also describe how precise control of temperature, pressure and sample thickness is achieved in these setups, and illustrate their performance with representative examples of IR and THz measurements on water and ice under diverse thermodynamic conditions.

## 2. Optical configurations

**Figure 2:**
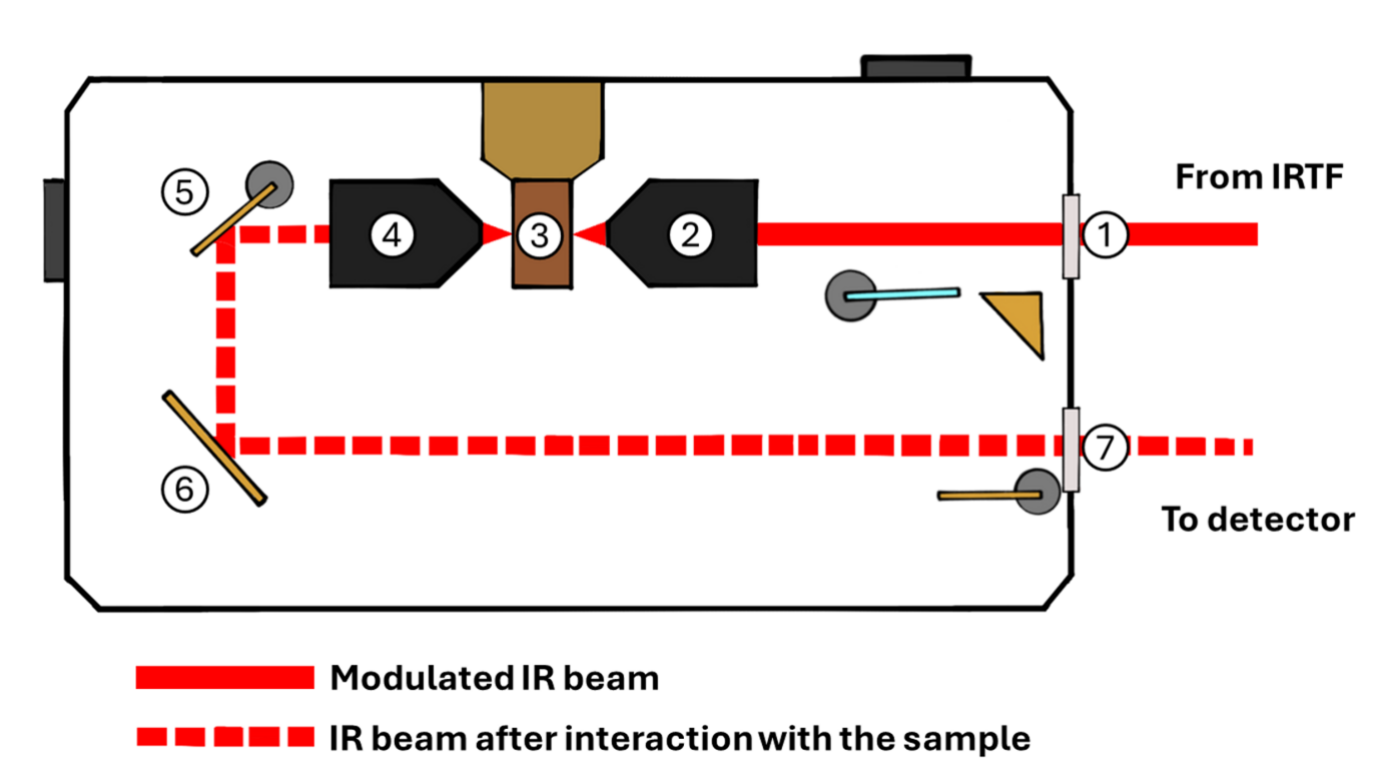
Components layout of the optical setup box in transmission configuration: (1) entrance of the IR beam and (7) exit towards the detector, (2) and (4) Schwarzschild objectives, (3) sample support, (5) flip mirror and (6) fixed mirror.

To enable measurements on sub-millimetric samples, all configurations employ two Schwarzschild reflecting objectives, each comprising a two-mirror Cassegrain-type system (Beck Optronic Solutions Model 5002, 15× magnification, numerical aperture 0.5, working distance 23.2 mm), positioned on either side of the sample support (see components 2 and 4 in Fig. 2 ▸). These objectives enable tight focusing of the synchrotron beam, achieving a spot size of approximately 50 µm in the mid-infrared (MIR) range and up to 200 µm in the terahertz/far-infrared (THz/FIR) range. The minimum size of the beam depends on the wavelength range explored. Following the diffraction limit, these values respect *d* > 1/2ν.

Each Schwarzschild objective is mounted on a motorized translation stage allowing *in situ* adjustment of the focus during experiments. The ensemble of the optics described in the following is placed inside a vacuum chamber, allowing a vacuum at 2 × 10^−6^ mbar during the experiment. The motorization of the optical elements and the connection of the chamber to the spectrometer is described in our previous paper (Voute *et al.*, 2016 ▸). Below, we briefly remind how it is operated, allowing measurements in transmission and reflection mode and the control of the sample cameras.

*Transmission configuration.* To measure the IR/THz transmission of a sample, the configuration shown in Fig. 2 ▸ is used. The incoming beam enters the optical setup chamber (1) and is focused onto the sample (3) by the first Schwarzschild objective (2). After passing through the sample, the transmitted light is recollimated by the second Schwarzschild objective (4). The collimated beam is then reflected sequentially by the flip (5) and fixed (6) gold-coated mirrors, directing it towards the exit port (7), and hence to the detector.

**Figure 3:**
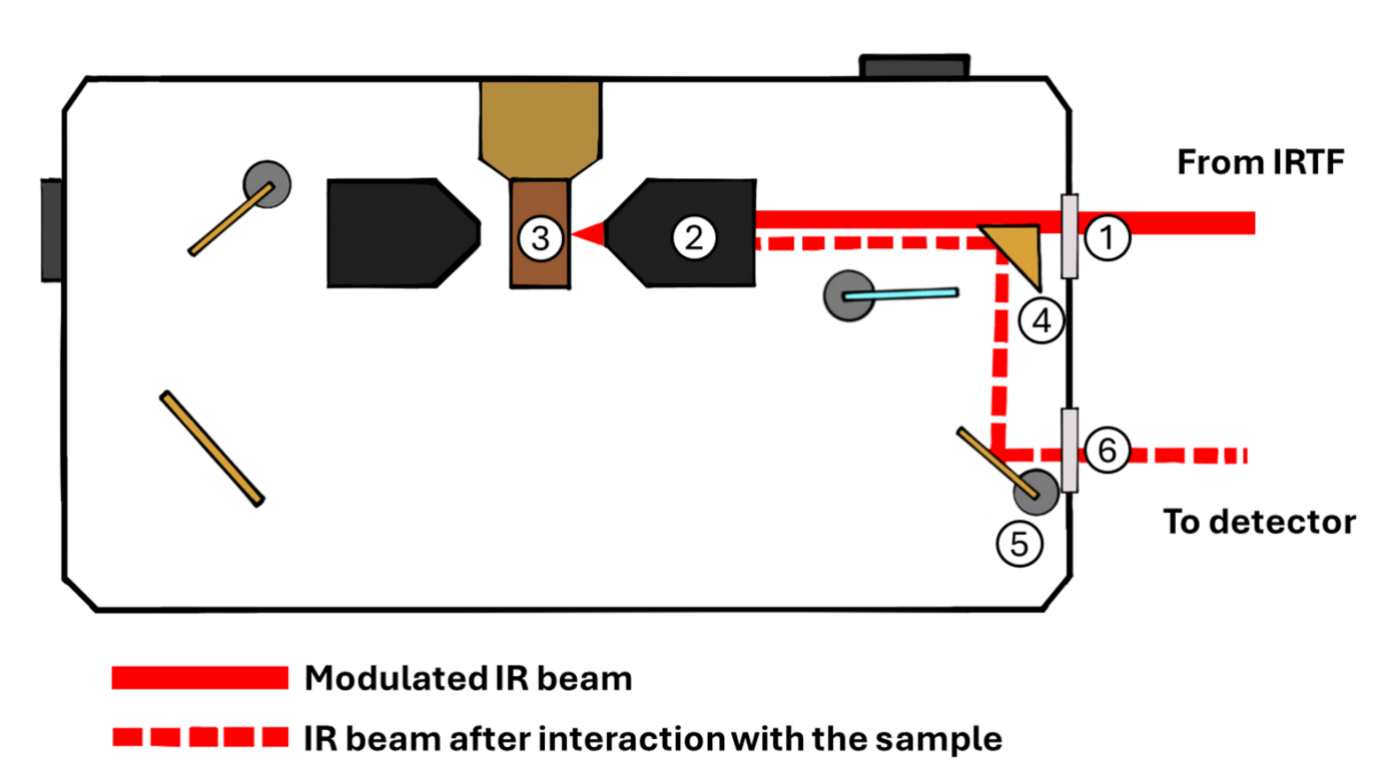
Components layout of the optical setup box in reflectivity configuration: (1) entrance of the IR beam and (6) exit towards the detector, (2) Schwarzschild objective, (3) sample support, (4) right angle mirror and (5) flip mirror.

*Reflectivity configuration.* For reflectivity measurements, the optical setup slightly differs, using only one Schwarzschild objective, as shown in Fig. 3 ▸. A knife-edge right-angle mirror (4) is inserted in the path of the incoming beam letting half of its cross-section go through the first Schwarzschild objective (2) which focuses it onto the sample (3). The light reflected by the sample is recollimated by the same objective and collected by the reflective surface of the right-angle mirror (4). It is then directed towards the gold-coated flip mirror (5), which reflects the beam to the exit port (6), and hence to the detector.

**Figure 4:**
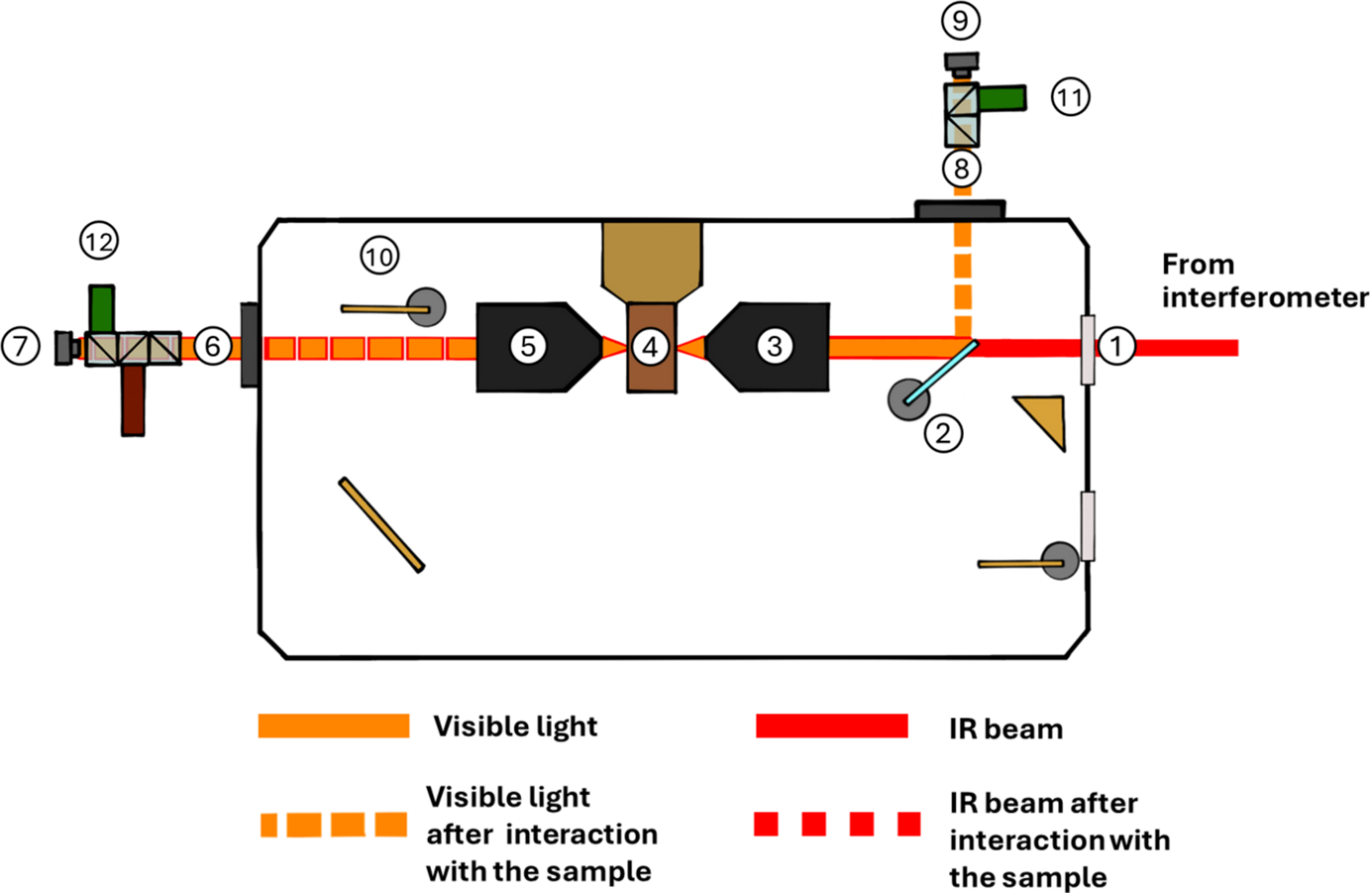
Components layout of the optical setup box during sample view configuration: (1) entrance of the synchrotron radiation, (2) movable beamsplitter, (3) and (5) Schwarzschild objectives, (4) sample support, (10) flip mirror, (6) and (8) viewports, (7) and (9) cameras, (11) and (12) visible lamps.

*Sample view configuration.* Two cameras and two visible lamps allow imaging of the sample environment and control of its alignment with the focusing IR beam during the experiment as shown in Fig. 4 ▸. Cameras (7, 9) and visible lamps (11) and (12) are positioned in front of the viewports (6, 8) and used in combination with the movable beamsplitter (2) and flip mirror (10) to permit visual alignment of the beam onto the sample. We can (i) image the sample and carefully select the spot to measure and (ii) check the state of the sample at different moments of the experiment.

**Figure 5:**
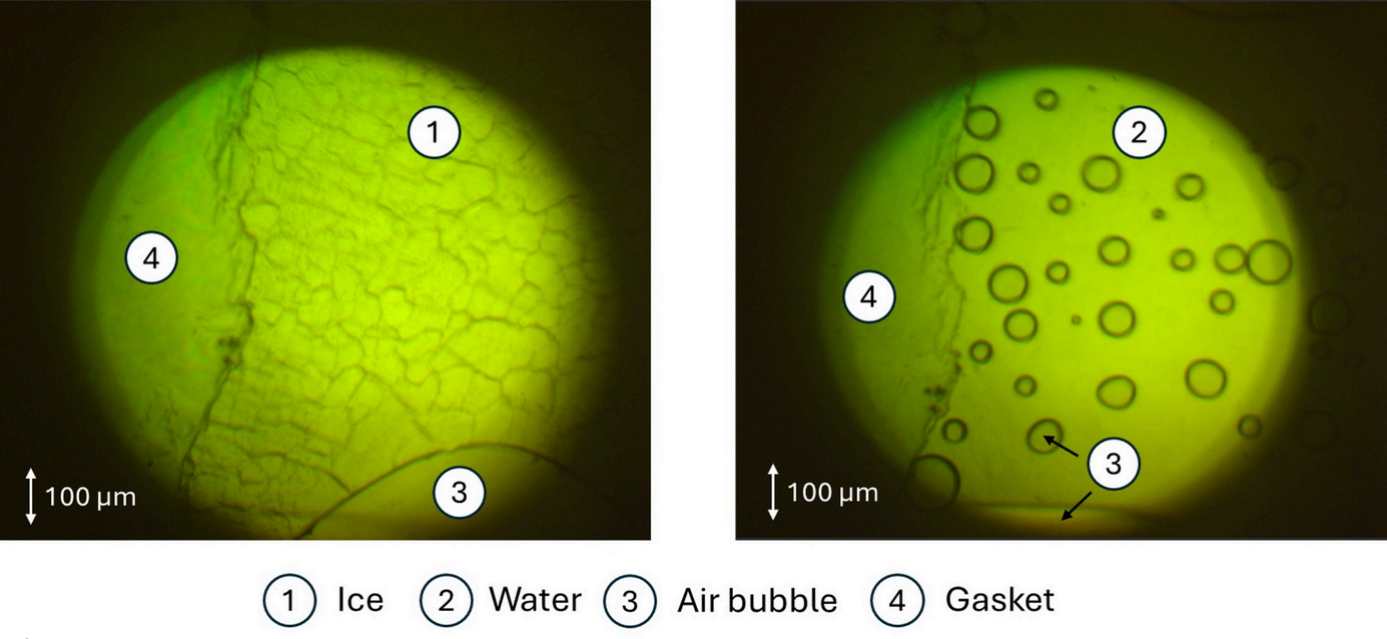
Photographs of a thin film of water at (*a*) 150 K and (*b*) 274 K. Different components can be distinguished, such as water in its liquid (1) and solid states (2), air bubbles (3) and part of the polypropyl­ene gasket (4).

An example is presented in Fig. 5 ▸, showing two photographs obtained during an experiment on a thin film of water at low temperature, where differences can be seen between water at 150 K, which has frozen into ice (left panel) and shows hexagonal microdomains, and liquid water after melting at 273 K (right panel).

## 3. Temperature control: heating and cryogenic systems

The optics described in the previous paragraph are duplicated in two optical setup boxes: one equipped with a cooling system (16–300 K) and the other with a heating one (room temperature to 600 K). One or the other can be connected to the IFS125 spectrometers at the AILES beamline according to the experiment’s needs. The two systems have been adapted to answer specific requirements, the principal being: (i) precise temperature control of the sample, (ii) compatibility with different sample holders and (iii) working under high secondary vacuum.

**Figure 6:**
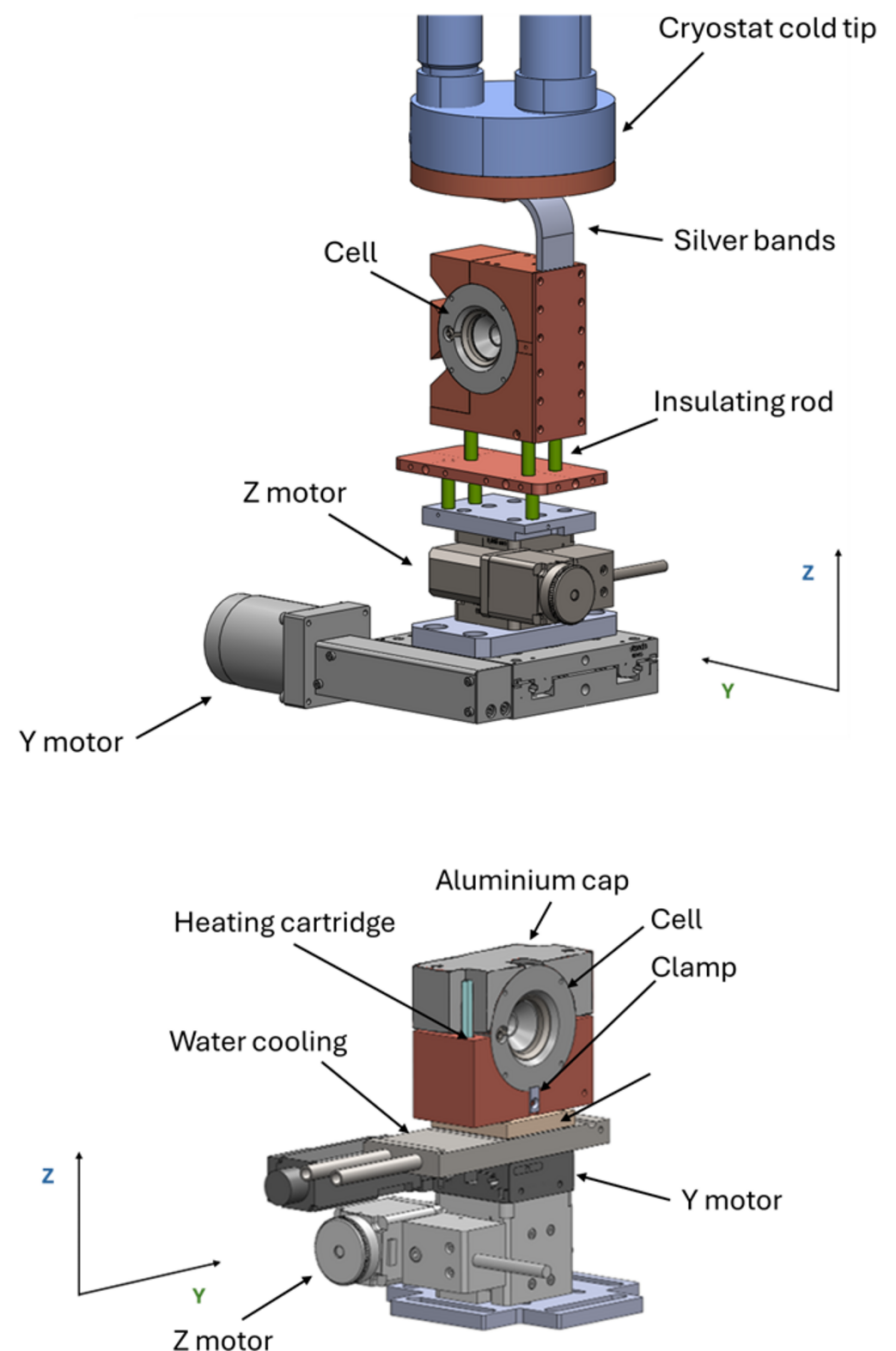
(Top) Cooling system and its main components, depicted without the external copper screen around the cell and a view of the lower part of the cryostat. (Bottom) Heating system and its main components.

For the cryogenic experiment, we use a Sumitomo cryostat (model SRP-O82 closed-cycle cryocooler), placed in a fixed position above the sample cell support. As illustrated in the top panel of Fig. 6 ▸, the sample cell is tightly enclosed in a holder made of copper placed on two motorized stages, which provide fine adjustments of the position along the *y* and *z* directions (*y* and *z* being, respectively, the horizontal and the vertical axes, perpendicular to the beam). The copper holder is mechanically decoupled from the 4 K cryostat cold tip and thermal connection is ensured by silver bands. While ensuring good thermal conduction, their flexibility (i) reduces the propagation of vibrations from the cold head of the closed cycle cryostat to the cell and (ii) allows repositioning of the cell under vacuum with the *z* and *y* motors, as thermal contraction of the cooled metal parts cause small displacements of the target point. In order to protect it from the room-temperature blackbody radiation from the chamber, the cell is surrounded by a thermal copper shield (not shown on the figure) connected to the 45 K intermediate temperature stage of the cryostat. This allows the cell body to reach temperatures as low as 16 K for the diamond anvil and uniaxial cells and 4 K for the liquid one.

For high-temperature experiments, the set-up is equipped with a heating cartridge directly inserted into the copper support of the sample cell, which is mounted on two motorized stages (bottom panel of Fig. 6 ▸). To prevent the sample cell from moving during the measurements and allow its reproducible positioning from one experiment to another, the sample cell is held on the semicylindrical copper support thanks to two metallic clamps and blocked from any further movement by an aluminium cap. To prevent the motors from overheating, a thermal insulator made of ceramic decouples them from the hot copper support. Furthermore, the motorized stages are attached to a water-cooled circuit. This system allows working under vacuum while precisely controlling the cell position.

## 4. Sample environments: cells and holders

To ensure maximum versatility, the setups have been designed to accommodate a broad range of sample cells, each tailored to specific experimental needs, whether probing bulk materials or liquids, under different conditions of pressure and temperature. All sample cells are integrated within a modular, thermally coupled sample holder, which ensures both precise optical alignment and efficient thermal transfer. Sample holders answer a precise experimental need that will be described below, along with their characteristic measurements.

### 4.1. Sample holders for optical studies under hydro­static pressure: diamond anvil cell

**Figure 7:**
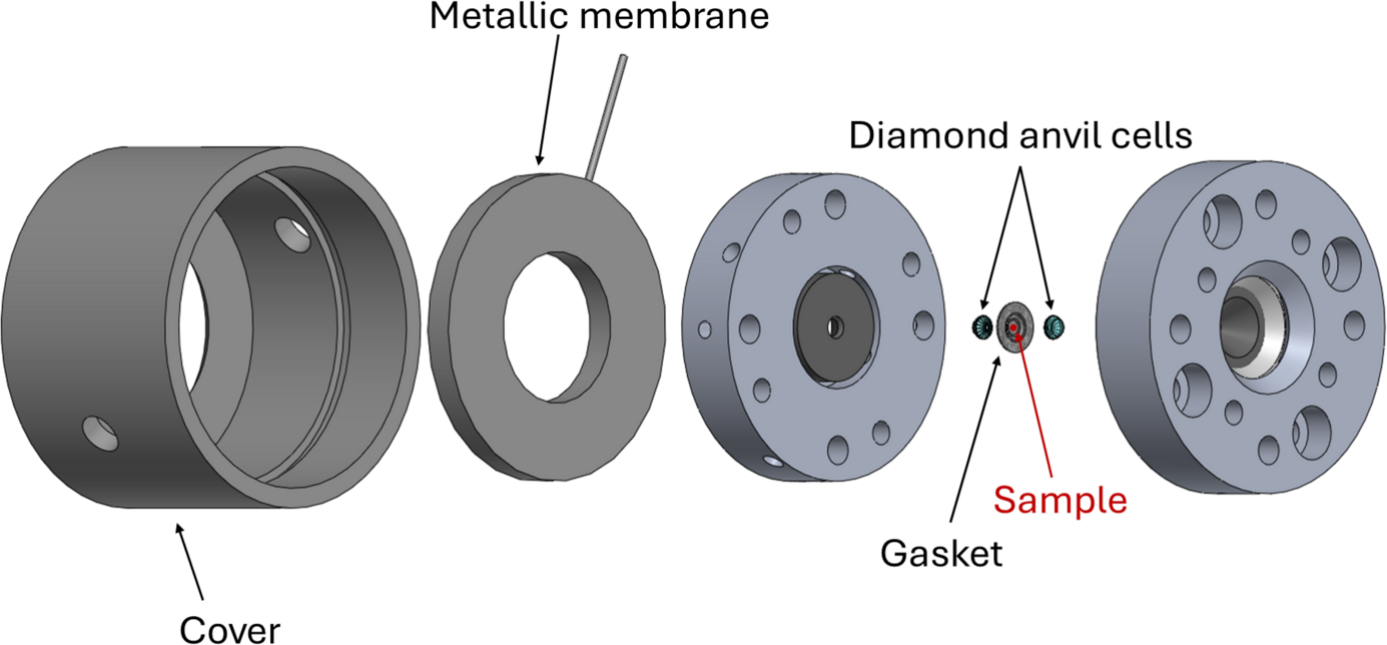
Schematic view of the diamond anvil cell main components.

High-pressure measurements are carried out using membrane-driven diamond anvil cells (MDACs), such as those developed by BETSA (Fig. 7 ▸). Each cell features two diamond anvils facing each other. The sample is centred between the two diamond-anvils and held in place by a pre-indented and holed gasket. The pressure on the sample is generated by pushing the anvils against each other. By inflating a metal membrane placed at the back of an anvil holder with an inert gas (helium or nitro­gen up to 110 bar), a force is propagated to the anvil, thus generating pressure at the sample location. The gas pressure inside the membrane is regulated by an Automatic Pneumatic Drive System (General Electric). The diamond anvils used are of type IIa diamond, selected for their IR transparency and mechanical robustness, with culets diameter between 100 µm and 2 mm. The size of the culets, but also the nature of the gasket and the diameter and thickness of the hole, will determine the maximum pressure reached by the sample (up to a maximum of 100 GPa with 100 µm culet diameter). Moreover, depending on the spectral region (THz/FIR, MIR or near-infrared) and pressure requirements, various pressure-transmitting media can be employed (*e.g.* KBr, polyethyl­ene or inert gas), see for example (Verseils *et al.*, 2023 ▸). The DAC is compatible with both the high and low temperature setups, which allows systematic investigation of materials research, including phase-diagram mapping and structural characterization. This means that the critical parameters to monitor during spectroscopic measurements are the pressure and temperature conditions applied to the sample.

**Figure 8:**
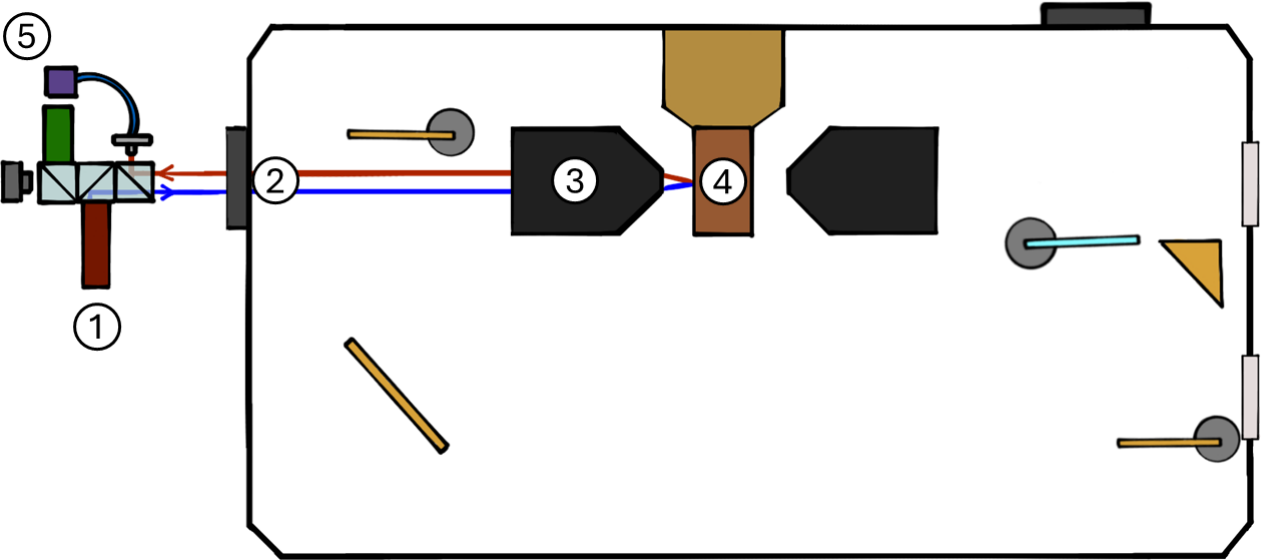
Schematic view of the ruby fluorescence measurement: (1) blue laser, (2) viewport, (3) Schwarzschild optics, (4) cell and (5) detector.

**In situ* pressure measurement.* The pressure within the sample can be monitored *in situ* by exploiting the pressure dependence of the fluorescence spectrum of ruby in the visible range. In practice (Fig. 8 ▸), a ruby chip, loaded with the sample in a pressure-transmitting medium, is illuminated by a blue laser (λ = 405 nm) (1) through a viewport (2). The emitted fluorescence is collected back through the same viewport and analysed using a visible-light Ocean Optics HR4000 spectrometer (5). A LabVIEW program, developed on the beamline (Voute *et al.*, 2016 ▸), fits the fluorescence lines in real time, providing a direct measurement of the pressure. Specifically, the wavelength shift and intensity ratio of the ruby fluorescence emission lines are used to evaluate the pressure and temperature inside the cell (Mao *et al.*, 1986 ▸; Datchi *et al.*, 2007 ▸).

**Figure 9:**
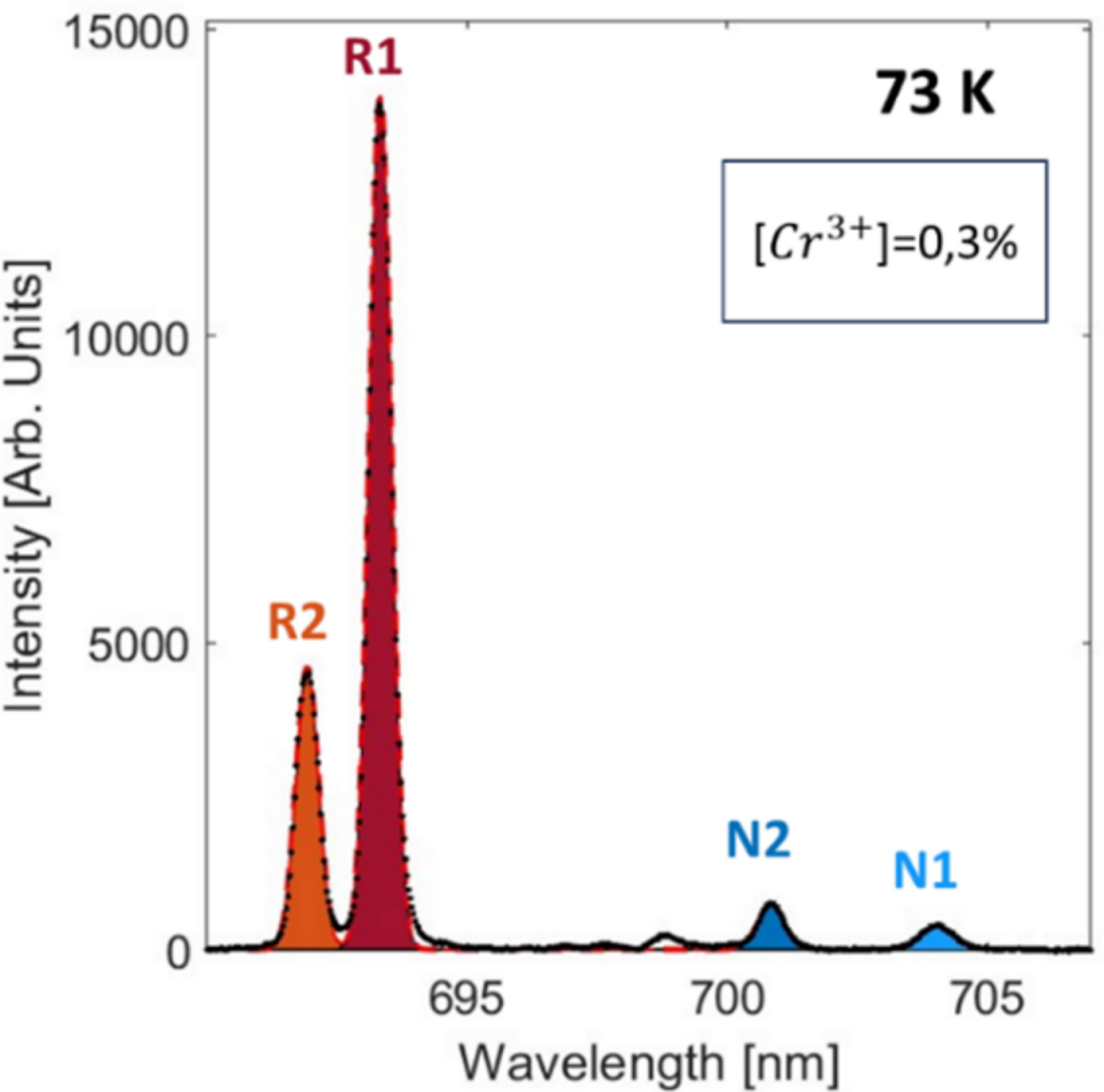
Fluorescence emission spectrum of a commercial ruby at 73 K. The orange and red areas correspond to Gaussians fitting the *R*_2_ and *R*_1_ lines, respectively, while the blue and light blue areas correspond to Gaussians fitting the *N*_2_ and *N*_1_ lines, respectively.

As shown in Fig. 9 ▸, ruby (Cr^3^^+^ in Al_2_O_3_) shows two main contributions on the fluorescence emission spectrum: the so-called *R*-lines (*R*_1_ and *R*_2_) due to transition in the single Cr^3^^+^ ions system and the *N*-lines due to transition in paired Cr^3^^+^ ions, thus their intensity will depend on the Cr^3^^+^ concentration. *N*_1_ and *N*_2_ bands are the strongest *N*-lines, produced by the second nearest-neighbour ions and the fourth nearest-neighbour ions, respectively (Powell *et al.*, 1967 ▸). Both the *R*- and *N*-bands shift in frequency and change intensity with pressure (Mao *et al.*, 1986 ▸) and temperature (Datchi *et al.*, 2007 ▸) and can be used to evaluate the pressure and temperature applied on the sample. However, commercial BETSA rubies are lightly doped ([Cr^3+^] = 0.3%), resulting in *N*-line intensities that are significantly lower than *R*-line intensities. Due to the lower precision of *N*-lines, only *R*-lines are used as pressure and temperature probes.

Although ruby is widely employed for pressure measurements, its use at high temperature is more imprecise. In fact, the intensity of the *R*-lines rapidly decreases with temperature, while the *R*_1_ and *R*_2_ lines broaden and become an unresolved band above ∼550 K (Datchi *et al.*, 2007 ▸). An alternative is to monitor the pressure shift of the ^5^*D*_0_–^7^*F*_0_ fluorescence line of SrB_4_O_7_:Sm^2^^+^ powder (685.41 nm at ambient conditions). The bandwidth of this fluorescence line increases slowly with temperature and pressure, allowing precise estimation of pressure up to 900 K (Datchi *et al.*, 2007 ▸). The laser system used to obtain fluorescence spectra of SrB_4_O_7_:Sm^2+^ powder is the same as for Cr-doped Al_2_O_3_ rubies.

### 4.2. Sample holders for optical studies under uniaxial pressure: uniaxial cell

The Razorbill Instruments CS2X0T uniaxial strain cell is a specialized device designed for applying tunable, homogeneous uniaxial strain to millimetre-sized samples in cryogenic and high magnetic field environments, with a particular focus on transmission and diffraction experiments. Featuring a sample access cone at the rear, the uniaxial cell enables unobstructed beam access for optical measurements, making it ideal for synchrotron setups. It employs piezoelectric stacks arranged symmetrically to apply tensile or compressive strain, while a flexure-constrained mechanism ensures homogeneous deformation without shear. A built-in capacitive displacement sensor provides real-time strain monitoring; knowing Young’s modulus of the sample the size displacement can be deduced (0.1 nm to 10 nm). The cell supports a maximum displacement of ±11 µm at cryogenic temperatures and a maximum force of ±50 N. Samples are mounted between titanium or beryllium–copper plates using ep­oxy resin, which is suitable for various types of samples, including bulk crystals, thin films and 2D materials. Adjustment allows for installing samples of section between 3 and 9 mm.

**Figure 10:**
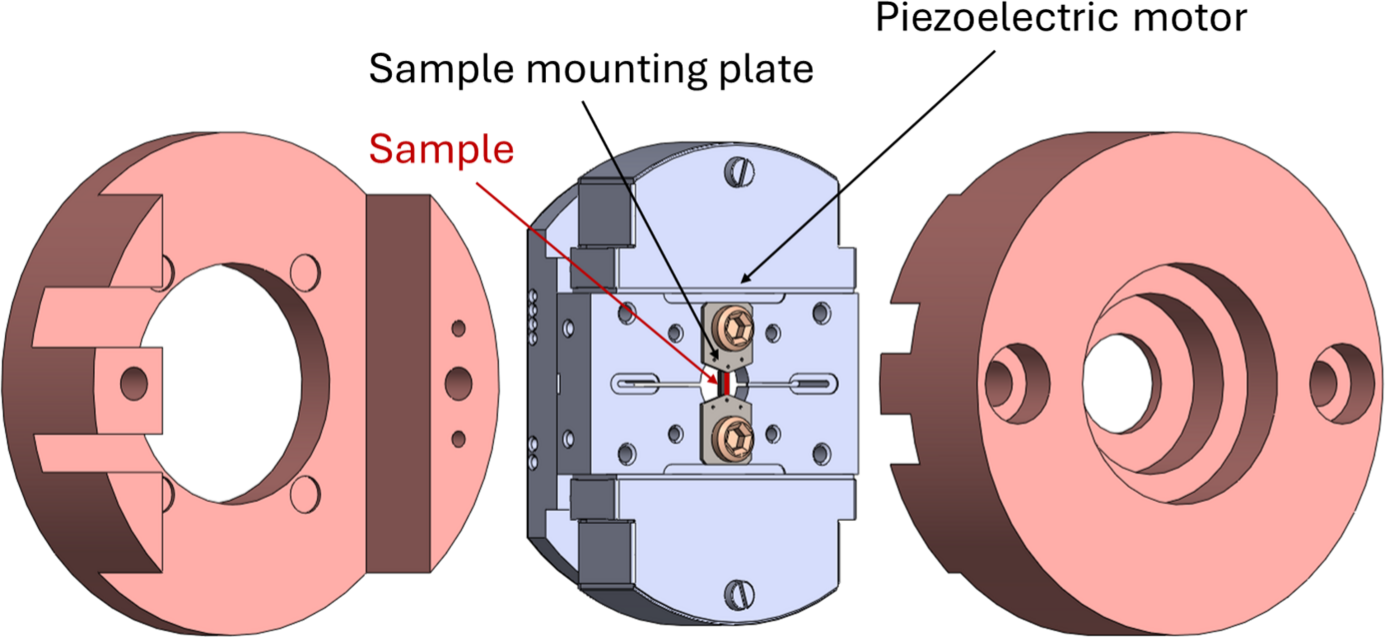
Schematic view of the uniaxial cell main components.

To ensure mechanical stability for the newly developed cell within our cryogenic system, two bespoke copper components were designed. Upon assembly, as illustrated in Fig. 10 ▸, these components enable efficient thermal conduction between the cryostat and the sample, maintaining operational performance over a temperature range from 16 K to 325 K. Furthermore, the chamber was equipped with specialized feedthroughs to facilitate precise control of the uniaxial cell. These include interfaces for optical fibre access, electrical cabling for motor actuation, and capacitance measurement of the system under mechanical strain.

### 4.3. Sample holder for optical studies of liquids at controlled temperature: liquid cell

**Figure 11:**
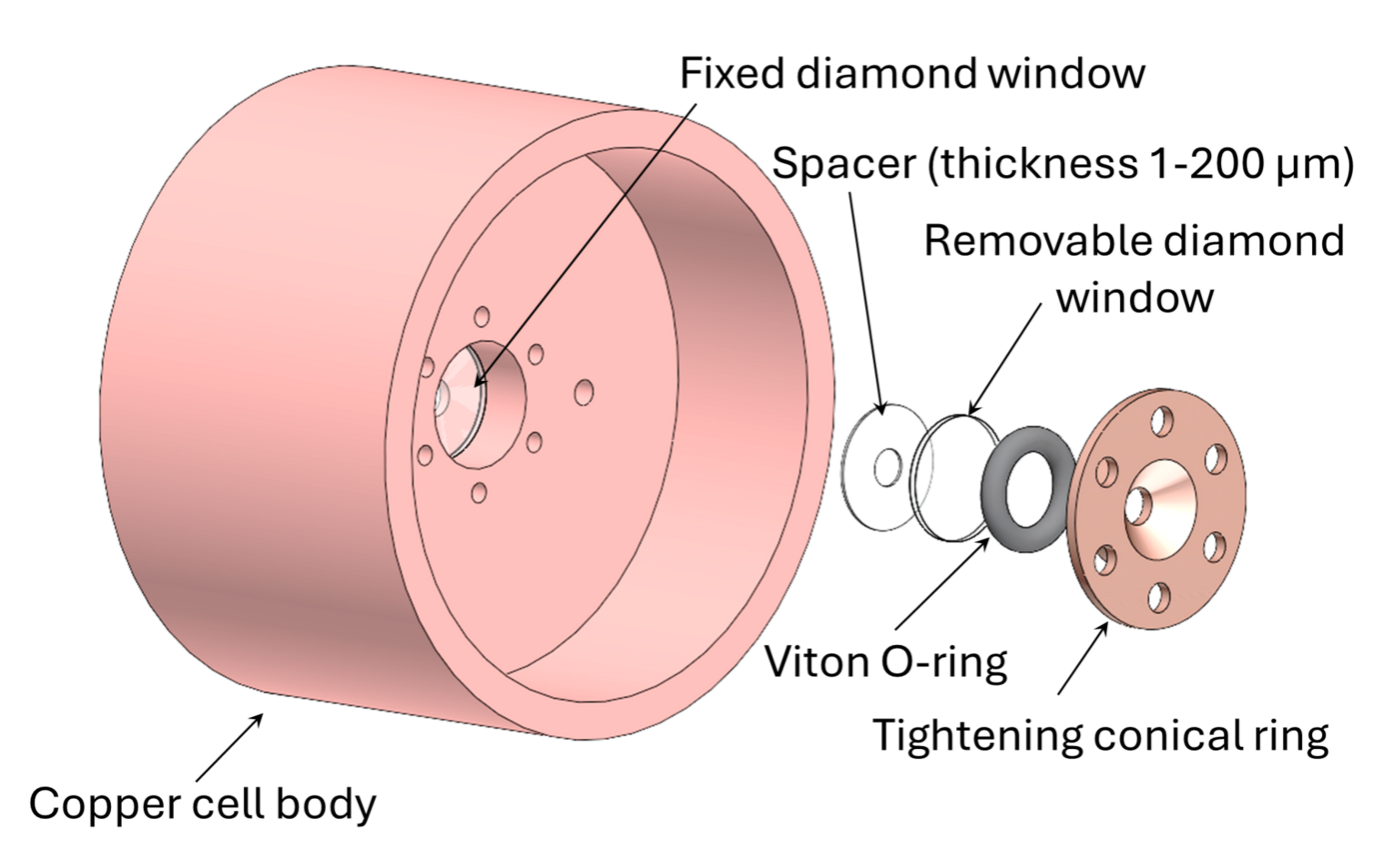
Schematic view of the liquid cell main components.

We designed a transmission cell for studies of thin films of water or other liquids under controlled temperature and sample thickness (Fig. 11 ▸). The requirements for this cell were driven by the need for measurements of absolute values of absorbance from water transmission spectra as a function of temperature at ambient pressure, in the context of satellite missions for atmospheric sensing (Palchetti *et al.*, 2020 ▸; Crevoisier *et al.*, 2014 ▸). Within this cell, a few microlitres of liquid are placed between two flat diamond windows held in a copper cell body. The thickness of the sample is defined by using a spacer (pierced disks) in polypropyl­ene of variable thickness (from 1 to 200 µm). To avoid signal saturation in case of highly absorbing liquid, the sample thickness needs to be as small as 0.5 µm, which can be attained without using a spacer. As an example, such thin films are needed when measuring water ice in the highly absorbing OH stretching region around 3200 cm^−1^. Notice that the sample thickness is the domineering factor in determining the absolute absorbance, and consequently the absolute absorbance precision varies between 2 and 5%. Indeed, as described further, the thickness is determined with a precision of 2% for the thicker layers and reaches 5% for thinner ones. The tightness of the cell is achieved using a temperature-resistant Viton O-ring pressed on one window using a conical ring screwed on the cell. The other diamond window is sealed on the copper body of the cell with silver conductive Ep­oxy (Epo-TEK from Pelco) for vacuum tightness and good thermal conductivity towards the sample.

One critical feature of this cell is its ability to maintain a hermetically sealed environment without applying external pressure on the sample. For the liquid cell, to reduce the pressure on the sample, the diameter of the window was designed to be as large as possible (10 mm). In addition, intentional introduction of air bubbles (see Fig. 5 ▸) within the liquid accommodates the volumetric expansion upon freezing. Consequently, the sample does not experience external pressure throughout the freezing process, both at room temperature and after ice formation, as proven in detail in Section 5.

**Figure 12:**
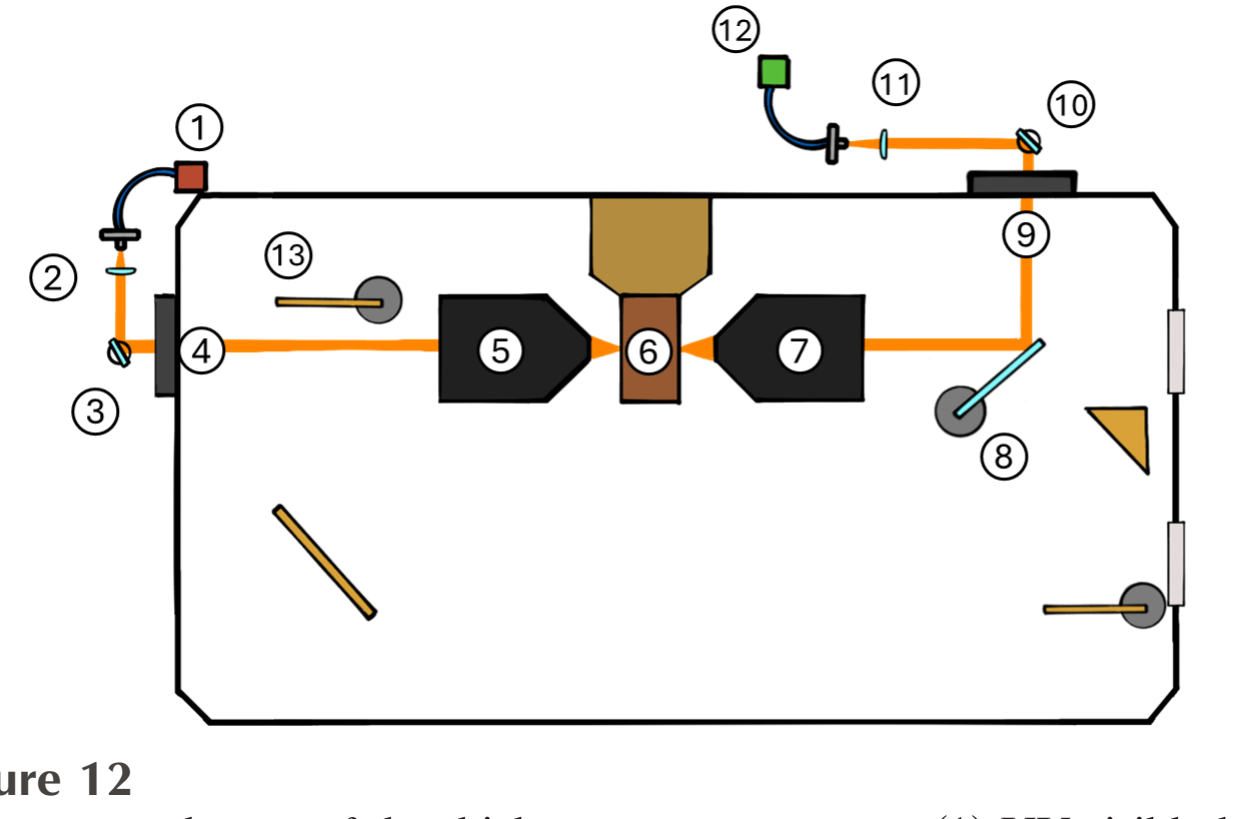
Components layout of the thickness measurements: (1) UV-visible lamp, (2) collimating lens, (3) and (10) manual flip mirrors, (4) and (9) viewports, (5) and (7) Schwarzschild objectives, (6) sample support, (8) beamsplitter, (11) focusing lens, (12) detector, (13) extracted flip mirror.

**Figure 13:**
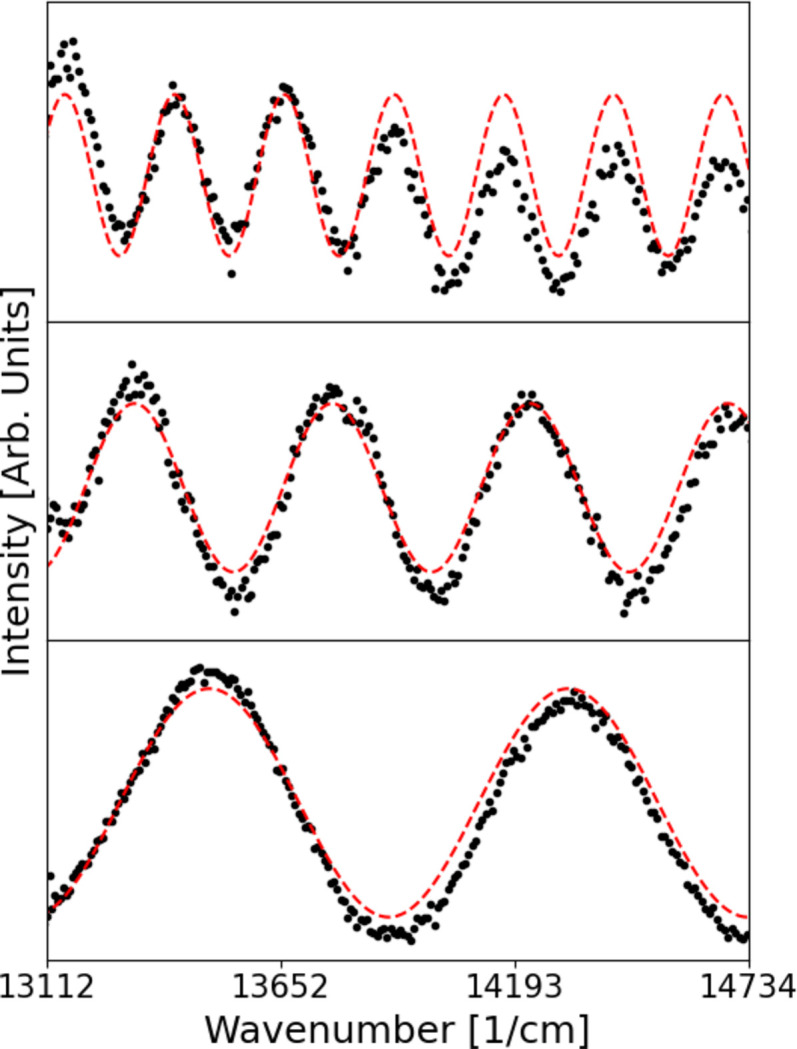
Typical fringes obtained shining UV-Visible light on a water sample (black dots) and corresponding sinusoidal fit (dashed red line) obtained using equation (1). The fringes from top to bottom correspond to water film thicknesses of 16.0 µm, 8.6 µm and 5.4 µm, respectively. Here the uncertainty on the sample thickness determination was close to 2% for all three samples.

**In situ* thickness measurement.* In the limit of the Beer–Lambert law, the extraction of the absolute value of absorbance requires normalization by the thickness of the sample. For this purpose, we take advantage of the interference fringes caused by multiple reflection of UV-visible light between the two parallel faces of the diamond windows of the cell and a sufficiently transparent sample. The optical box layout for thickness measurement is depicted in Fig. 12 ▸. An external halogen-deuterium source (1) (range 10000–30000 cm^−1^) via an optical fibre shines UV-visible light towards a collimating lens (2). The parallel beam is then directed towards the viewports (4) thanks to a manual flip mirror (3). Meanwhile, the flip mirror (13) and the beamsplitter (8) are extracted and inserted, respectively, so that the light can pass through the cell and is directed towards the other viewport (9). Finally, the beam is reflected by a manual flip mirror (10) and focused by a lens (11) on the optical fibre connected to an OceanOptic FLAME-S-XR1-ES spectrometer (12). From this measurement, we obtain a series of sinusoidal oscillations (Fig. 13 ▸) of period *T* related to the sample thickness *d* and (real) refractive index *n* using the following relation,
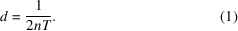
The UV-visible interference fringe method allows for precise *in situ* determination of transparent film thicknesses ranging from ∼0.3 µm to several hundred micrometres, depending on the sample’s refractive index. The minimum measurable thickness is limited by the spectral bandwidth of the light source and the spectral range of the spectrometer, while the maximum thickness is constrained by the spectrometer resolution. Using fringe-fitting routines, thickness values can be extracted with an uncertainty better than 5%, as illustrated by measurements on water films of 16.0 µm, 8.6 µm and 5.4 µm (see Fig. 13 ▸).

### 4.4. *In situ* temperature measurement for DAC and liquid cell

Just as thickness measurement is essential, accurately determining the sample temperature represents a critical step in high-pressure and/or extreme-temperature experiments. Therefore, it is common to use temperature sensors placed as close as possible to the sample, and, when possible, *in situ* measurements (*e.g.* ruby fluorescence) to determine the actual temperature of the sample. In the present DAC setup, the temperature is measured by a Cernox sensor connected to the outside of the cell’s metal body during the cooling/heating process. For example, titanium used for cell body (and even more so its alloy Ti-6Al-4V) of the DAC is a poor thermal conductor compared with copper. This implies that there is a temperature gradient between the body of the cell and the sample between the anvils, particularly during cryogenic or high-temperature experiments. Due to space constraints and the complexity of mounting the DAC cell, it is not possible to place a temperature sensor on the diamond when the sample is in place. Thus, in order to know the exact temperature of the sample during an experiment with the DAC, two methods can be used: (i) a correction of the temperature measured on the DAC’s body (using the calibration curve described below) or (ii) the temperature measured from fluorescence signal from a ruby chip placed inside the cell. Since the ruby fluorescence signal depends simultaneously on pressure and temperature, the latter method is more suited for experiments in which the temperature remains fixed while applying the pressure. The main advantage of this measurement is that it takes into account the thermal capacity of the specific sample. For all other pressure/temperature experiments, correction by calibration curve should be used instead.

**Figure 14:**
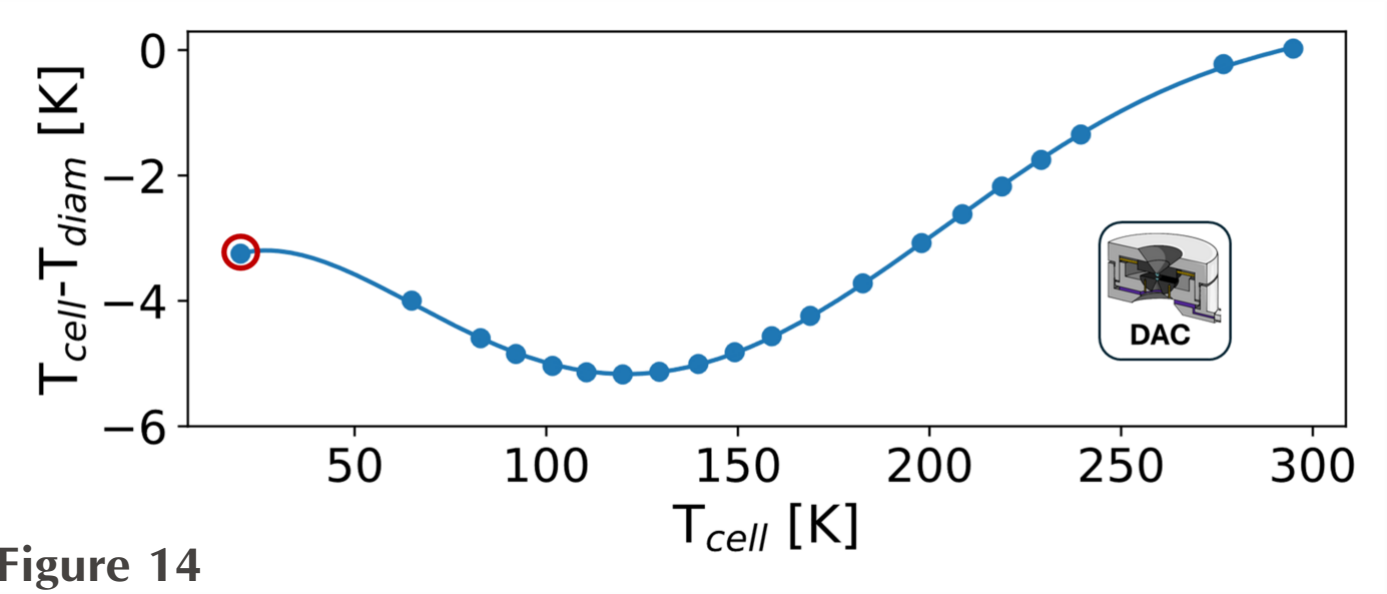
Temperature difference between the outer body of the DAC (*T*_cell_) and its diamond anvils (*T*_diam_) against read *T*_cell_. The circled measurement was obtained after a thermalization time longer than 20 min.

Fig. 14 ▸ represents the temperature differences in the DAC between the Cernox sensor placed on the body of the DAC (*T*_cell_) and a Cernox sensor glued on one diamond of the DAC (*T*_diam_). This gap is specific to the DAC and can be used to correct temperature measurements. Each measurement was taken after waiting 20 min for the cell to thermalize, except for the lowest temperature which was measured after several hours of thermalization. As can be seen in Fig. 14 ▸, the temperature inside the cell is always higher than the one measured outside the cell, due to the proximity of the cell body to the cold tip. This difference evolves from 3 K at the coldest point to ∼5 K down to 150 K and decreases towards zero when approaching room temperature.

**Figure 15:**
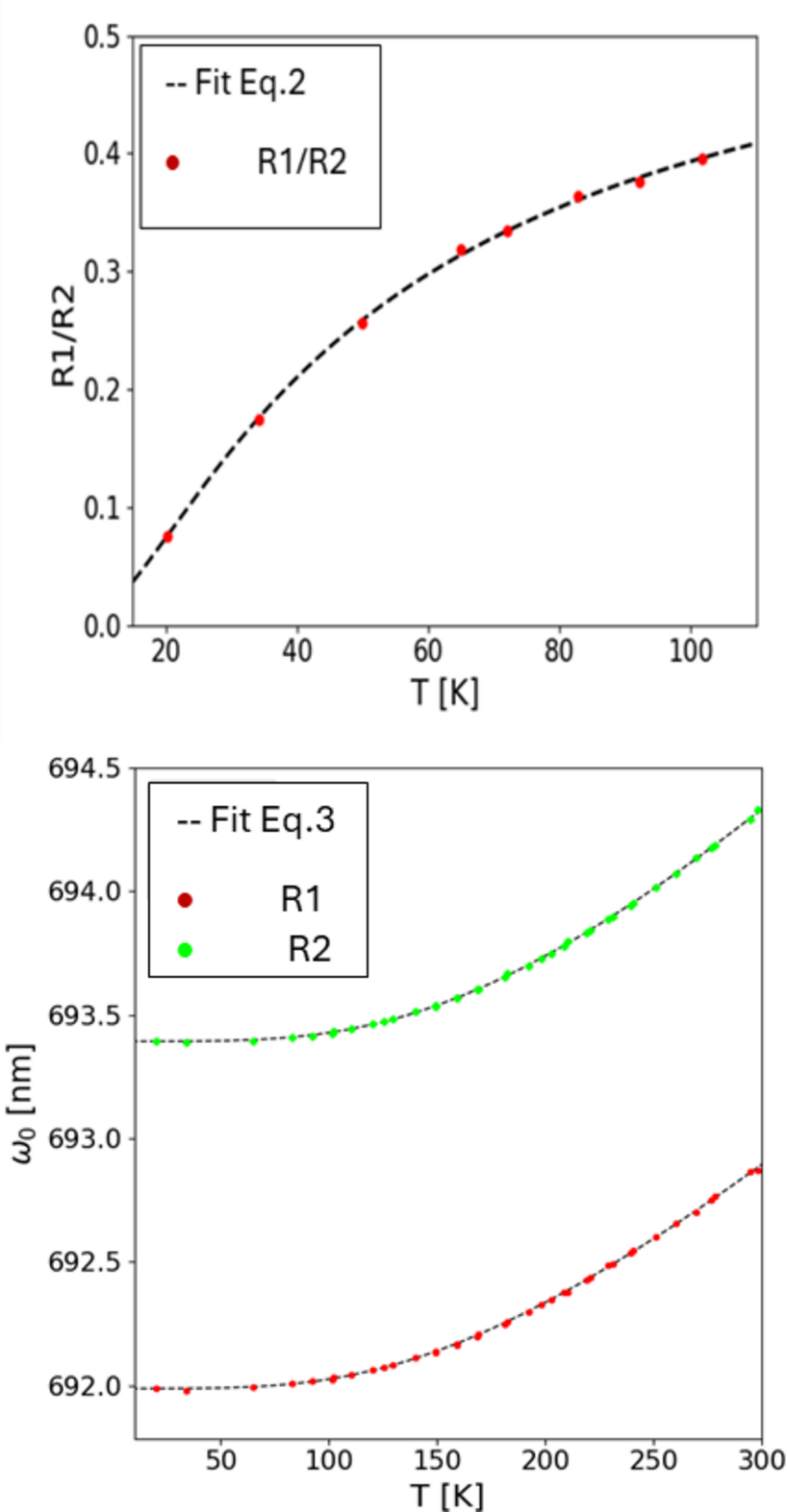
(Top) Temperature variation of the *R*_1_/*R*_2_ ratio between 20 and 100 K (red dots) and corresponding fit obtained using equation (2). (Bottom) Temperature variation of the *R*-lines peak position between 20 and 300 K. In green the values for the *R*_2_ line and in red for the *R*_1_, while in black the fitted curve obtained using equation (3) for the two sets of values.

Alternatively, the sample temperature can be determined *in situ* with the temperature-dependent positions and intensities of fluorescence lines of a ruby chip placed inside the DAC. For temperatures below 100 K, it is possible to use the ratio of the intensities of the *R*-lines (Weinstein, 1986 ▸) which can be described using Boltzmann statistics,
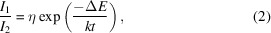
where η and Δ*E* are fitted parameters and represent the ratio of the quantum efficiency of the *R*_2_ to the *R*_1_ transition for*kT* > Δ*E* and the energy given by *R*_1_ and *R*_2_ splitting, respectively. For more details, see Weinstein (1986 ▸). The top panel of Fig. 15 ▸ shows the results of the fit of intensity ratios using equation (2) for measured temperatures between 20 and 100 K.

For higher temperatures, the electronic states start to interact with acoustic phonons, and equation (2) can no longer properly explain the variation of lines intensity. In this case, we use the frequency shift of the *R*-lines, as described by the McCumber and Sturge law (Mccumber & Sturge, 1963 ▸), 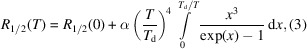
where *R*_*i*_(0) is the position of the *R*_1_ or *R*_2_ line at 0 K, α is the electron–phonon coupling constant and arises from the scattering of phonons by the impurity Cr^3+^ ion, and *T*_d_ is the Debye temperature. Moreover, since all phonons are not coupled to the electronic state, this *T*_d_ is not necessarily the same as that which determines the heat capacity and thus is one of the fitted parameters (Ragan *et al.*, 1992 ▸). The result of the fit obtained using equation (3) is shown in the bottom panel of Fig. 15 ▸.

**Figure 16:**
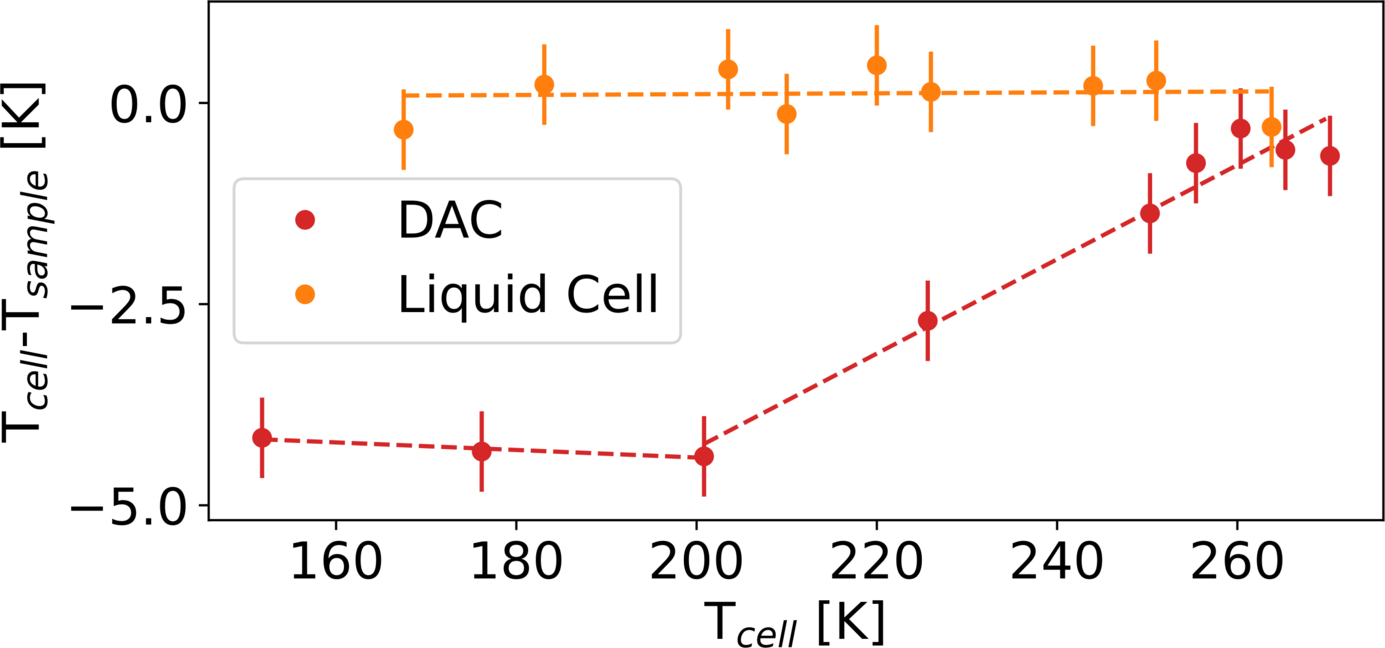
Temperature difference between the body of the cell (*T*_cell_) and that of the sample deduced from the ruby fluorescence (*T*_sample_) against *T*_cell_, for the liquid cell (orange) and DAC (red), with a water ice sample between 160 and 270 K.

This method has been used to evaluate the temperature difference between the cell’s body and the sample for two ensembles: the DAC and liquid cell. Both cells were filled with water sample and a ruby probe, serving as a reference for a sample temperature (*T*_sample_). As illustrated in Fig. 16 ▸, in the DAC the temperature discrepancy between the cell and the sample is about 4.2 K below 200 K, which then diminishes, approaching zero as the temperature increases. The observed temperature gap aligns with our earlier findings obtained with two Cernox sensors over the same temperature range. This proves the equivalence of the two solutions for a sample at fixed pressure and confirms the necessity of the temperature correction reported in Fig. 14 ▸ for all other experiments. On the other hand, the temperature difference between the cell and the sample in the liquid cell oscillates around zero, with a maximum deviation of 0.8 K, inside the margin of error of the measurements. These observations confirm the good thermal conductivity of the liquid cell. Thus, temperature correction is not necessary when using the liquid cell.

## 5. Setup performances: an illustration with FIR spectra of water ice

The phase diagram of ice is complex, mainly due to hydrogen bonding leading to different crystalline phases according to pressure and temperature. The crystallographic structures of ices were mostly determined in the mid-1980s (Kuhs *et al.*, 1984 ▸). Lowering the temperature leads to proton ordering whereas increasing the pressure reduces the distances between second shell neighbours. FIR spectroscopy directly probes the intermolecular modes, in particular the connectivity band (stretching vibration between two hydrogen-bonded water molecules) and the librational modes (hindered rotation of hydrogen-bonded water molecules). By studying the evolution of the connectivity band in temperature and pressure, we can address the nature of the hydrogen bonds, the structural changes and the proton ordering in water (Bertie *et al.*, 1968 ▸).

In this section we briefly present the FIR absorbance of water and water ice, formed at low temperature and ambient pressure [hexagonal ice, Ice Ih (Bertie, 1968 ▸)], under high pressure and low temperature [Ice VI (Bertie *et al.*, 1968 ▸)] and under high pressure and high temperature (transition between liquid and ice VI). They were measured using our new set-up combined with the liquid cell or using a DAC. Ruby chips were placed in the cell in order to evaluate the pressure inside the DAC and liquid cell. Measurements were performed in transmission in the FIR between 40 cm^−1^ and 700 cm^−1^, using the synchrotron beam with one of our IFS125HR spectrometers equipped with a 4.2 K bolometer and a 6 µm Mylar beamsplitter. For each spectrum, 400 scans at 2 cm^−1^ resolution were co-added.

### 5.1. Hexagonal ice (Ih)

**Figure 17:**
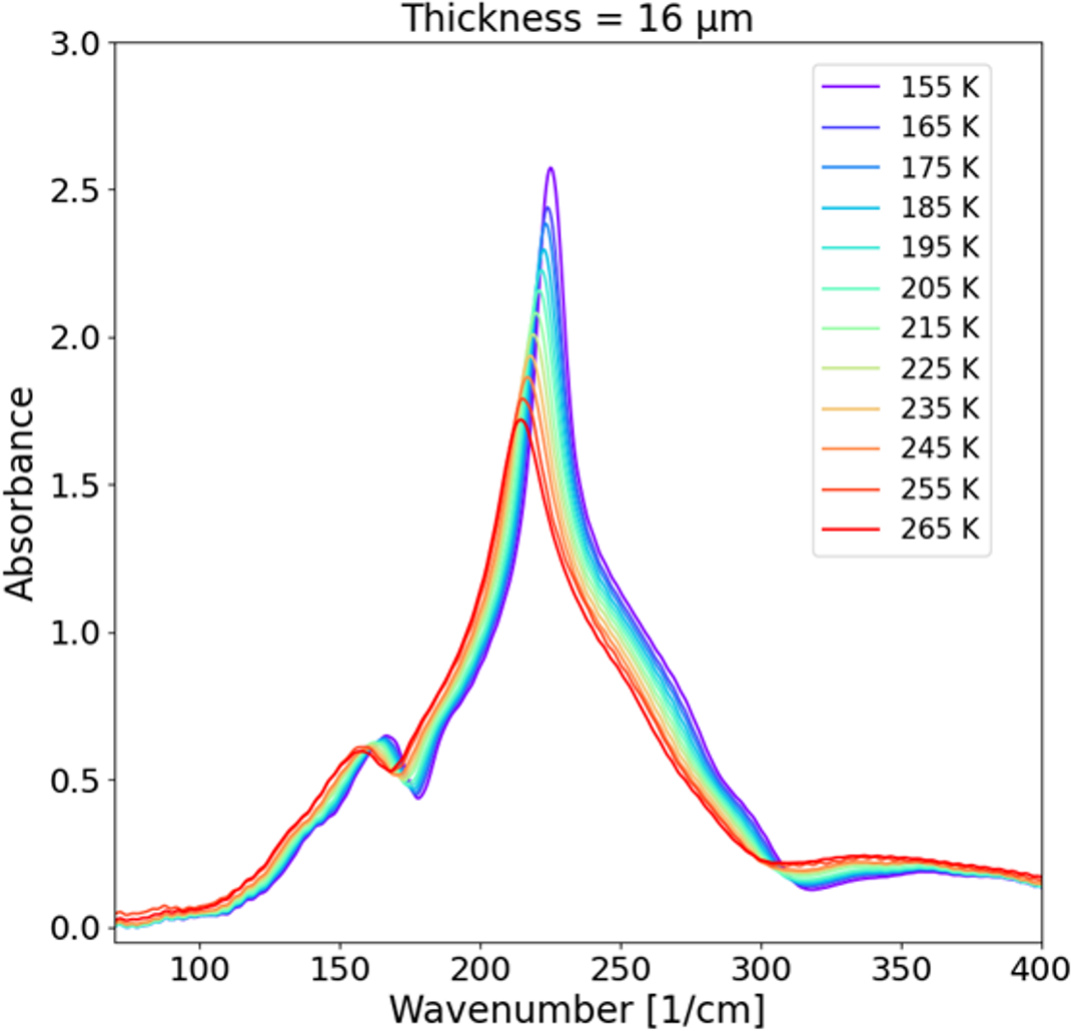
Far-infrared absorbance spectra of hexagonal ice between 155 (blue) and 265 K (red). The connectivity band peaks at 160 and 220 cm^−1^ blue shift and increases in intensity with decreasing temperature.

To obtain water ice at low temperature and ambient pressure we used the liquid cell with a 16 µm spacer. As detailed previously, the liquid cell prevents the sample from undergoing any external pressure, even after water expansion due to freezing, making it the most suitable cell for this type of study. By filling the cell with liquid water and freezing it between 155 and 265 K, we obtain crystalline ice in its most stable hexagonal phase [a proton disordered phase (Bertie, 1968 ▸)]. In Fig. 17 ▸, we can distinguish two main components of the connectivity band of hexagonal ice, at 160 cm^−1^ and 220 cm^−1^, both blue shifting and increasing in intensity as temperature decreases, a sign of the higher proton order developing.

During the measurements, we verified the absence of external pressure on the sample and estimated the temperature using the fluorescence of a ruby chip placed inside the water.

**Figure 18:**
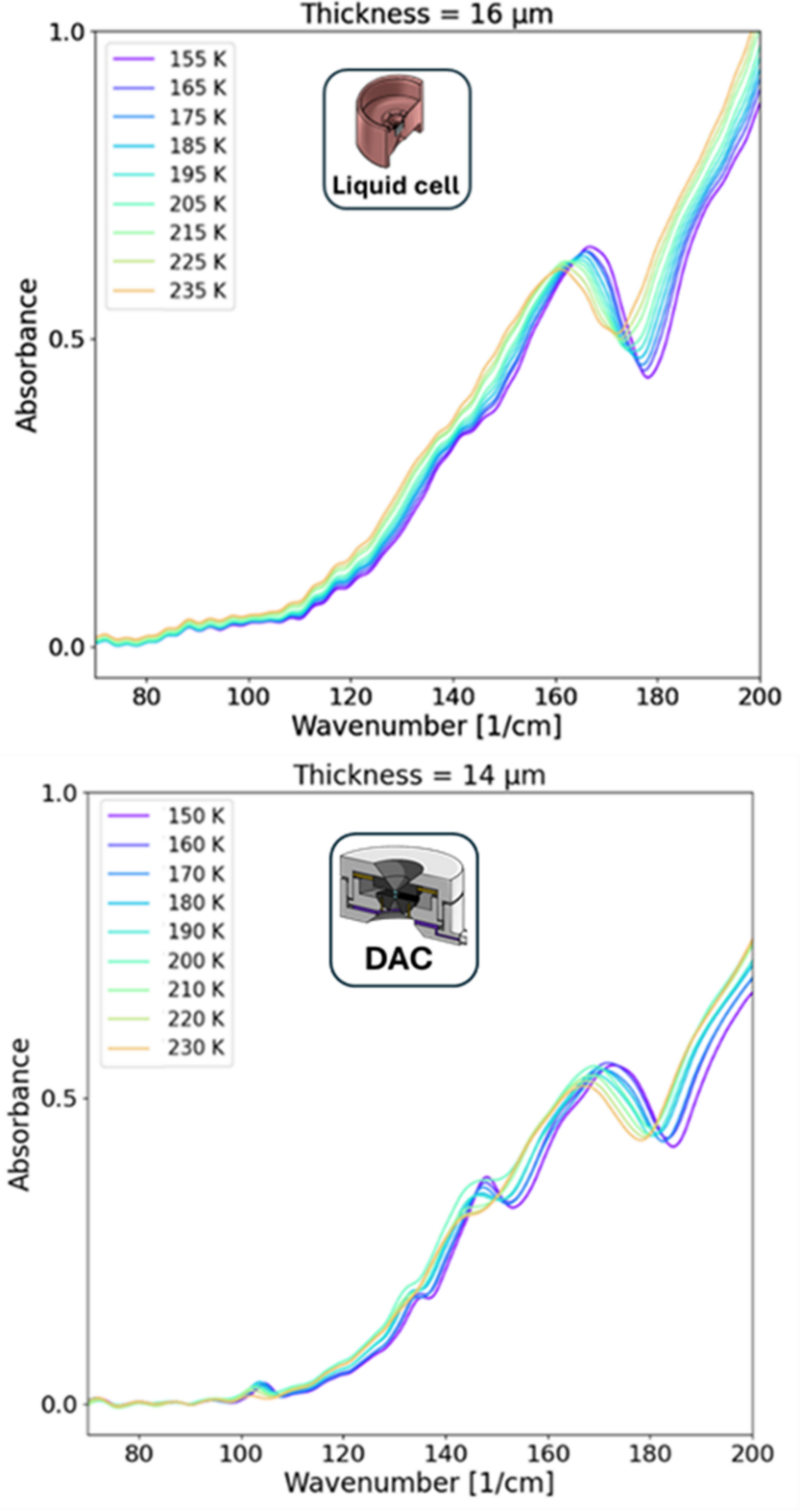
Comparison between the low energy peak of the connectivity band measured on water ice formed inside the liquid cell (top) and the DAC (bottom) in the temperature range 150–230 K. The presence of low intensity peaks for ice obtained in the DAC points at the presence of another form of ice different from the hexagonal one.

To stress the importance of using specific cells for different goals and further confirm the absence of external pressure in the liquid cell, we compared the results obtained using the liquid cell and the DAC with 2.1 mm culet diameter diamonds while following the same temperature cycle and without external pressure from the membrane. By comparing the ruby fluorescence line for the two cells, we observed that in the DAC a residual pressure of ∼20 MPa is always present upon closing even with large culet diamond. This can raise up to ∼200 MPa with water expansion due to freezing, changing the phase of ice formed. This is confirmed by comparing the spectral features of the connectivity band for the liquid (top panel of Fig. 18 ▸) and the diamond anvil cell (bottom panel of Fig. 18 ▸). The appearance of some low intensity peaks (106 and 150 cm^−1^) and the transformation of the 136 cm^−1^ shoulder into a peak at low temperature (between 150 and 230 K) for ice formed in the DAC are typical features of ice II (Bertie, 1968 ▸), proving that the water ice observed is not only hexagonal but a mixture of Ih and ice II, which can be formed in the presence of pressure above 100 MPa. As intended, the water in the liquid cell, on the other hand, does not experience additional pressure in the limit of our measurements through all the temperature cycle and leads to the formation of pure hexagonal ice.

### 5.2. Ice VI

**Figure 19:**
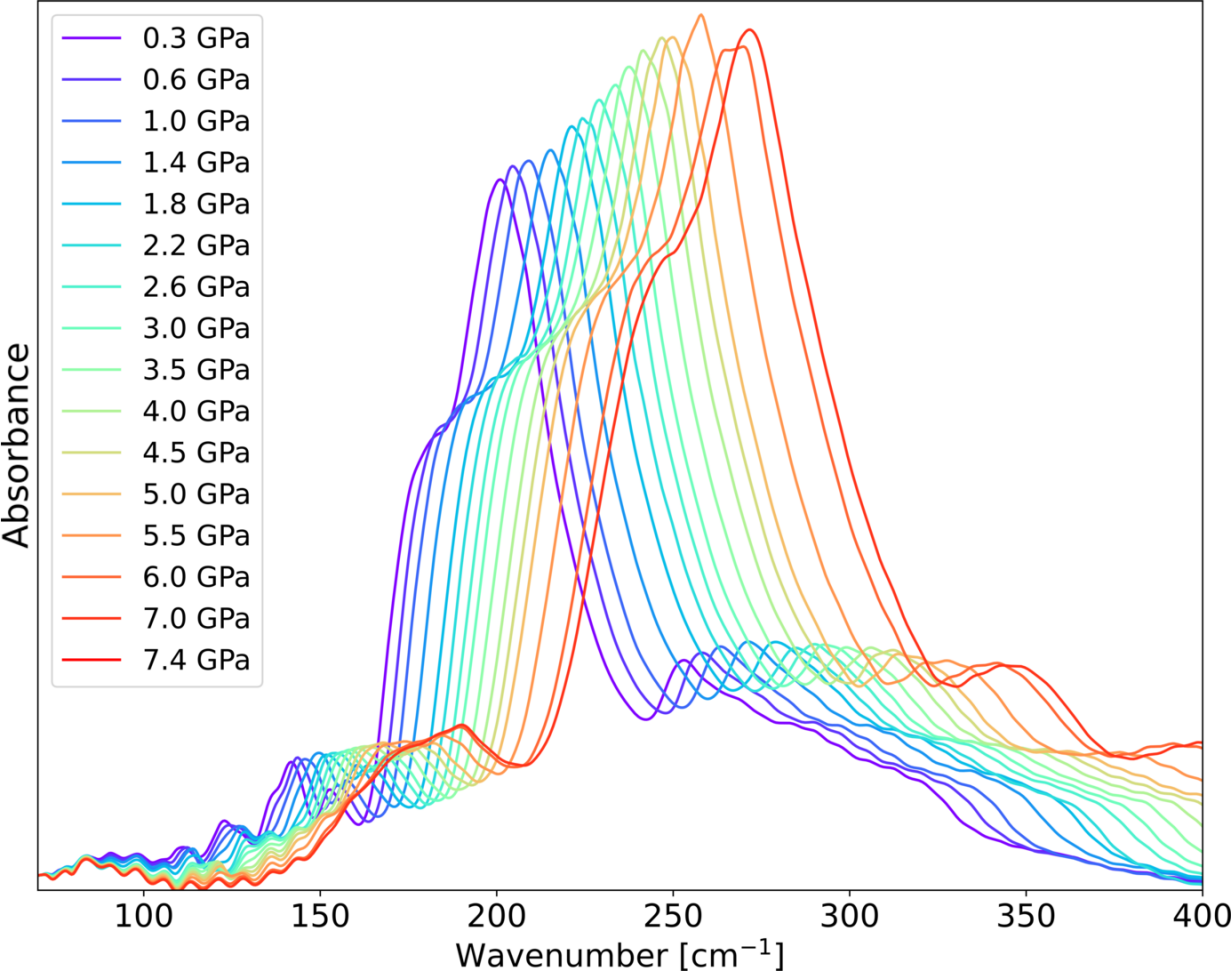
Absorbance spectra of ice VI at 50 K and pressure varying between 0.3 (blue) and 7.4 GPa (red). As expected, the connectivity band blue shift and increase intensity as the pressure increases.

Ice VI is a proton disordered phase of water that can be obtained at low temperature and high-pressure range (Dunaeva *et al.*, 2010 ▸). The broad absorption of the connectivity band (Fig. 19 ▸) is a signature of the orientational disorder of this ice phase. Nevertheless, ice VI features are still very distinct from other disordered phases, such as ice Ih shown previously. Ice VI is formed by two interpenetrating but not interconnected hydrogen-bond networks, where water molecules are arranged in a tetrahedral structure (Bertie, 1968 ▸). We monitored the evolution of this phase of water with pressure by using the DAC cell coupled with the cryostat. Spectra of ice VI at 50 K were obtained over the pressure range 0.3–7.4 GPa. We also observe the stability of this phase at 50 K, with pressure increase resulting in a blue shift and a more intense connectivity peak, which reflects the strengthening of the proton bonding in ice.

### 5.3. High temperature and high pressure: liquid–solid transitions

**Figure 20:**
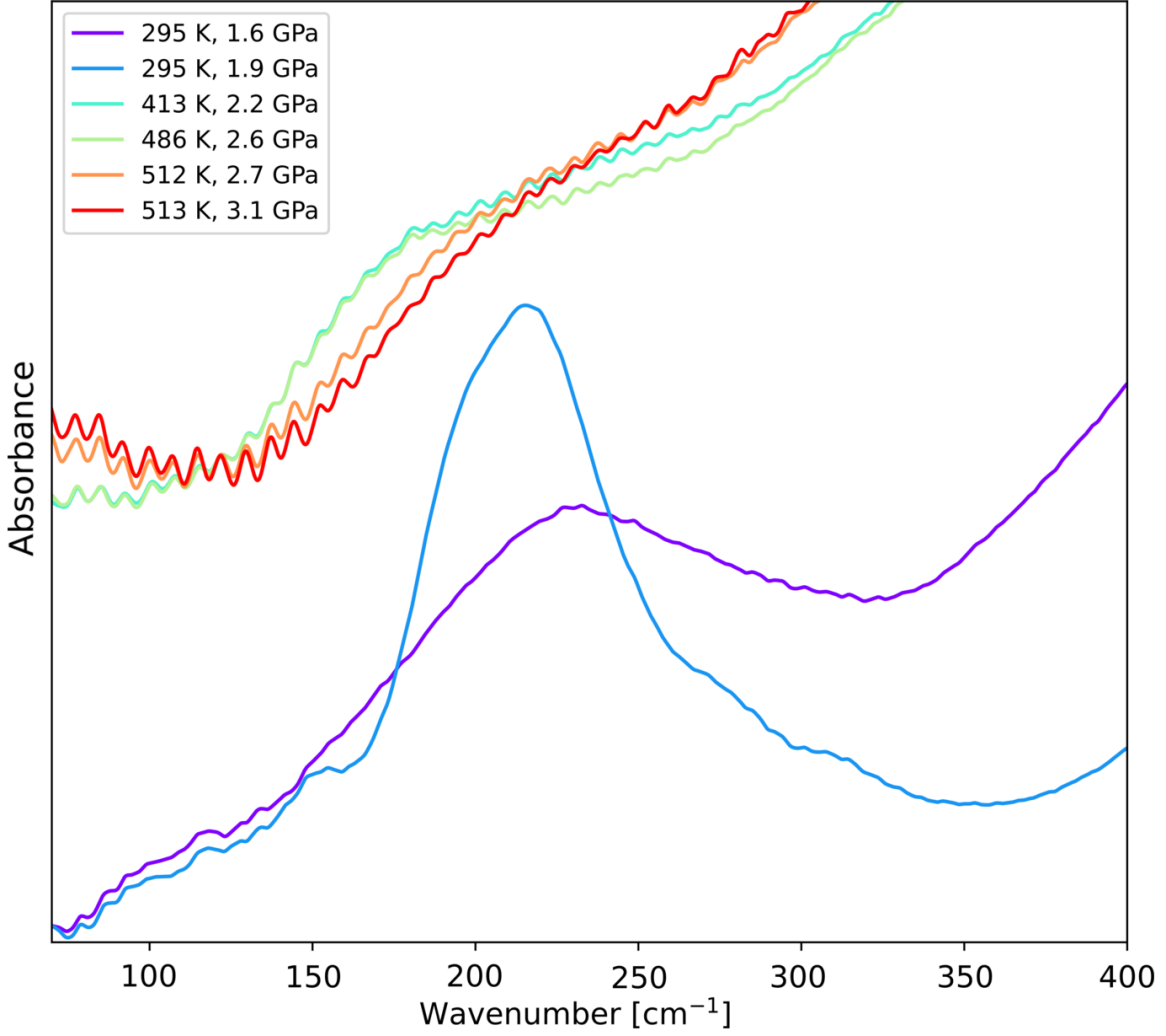
Absorbance spectra of water under different pressure–temperature (*P*–*T*) conditions. Starting from liquid water at room temperature and low pressure (violet line), compression of the sample leads to the formation of ice VI at room temperature and approximately 2 GPa (blue line). Upon heating and pressure increasing, liquid water is recovered in the temperature range 413–513 K.

The experimental setup can also be used to explore the liquid–solid transition of water under high-pressure and high-temperature conditions. By monitoring the connectivity band, direct information on the hydrogen-bond network can be obtained. Significant changes in this band indicate structural modifications in water: in the liquid phase, the connectivity band is broad, whereas it becomes narrower upon transition to ice VI due to the increased molecular ordering in the solid phase (Fig. 20 ▸). After the solidification of water at ∼2 GPa and ambient temperature, the liquefaction is recovered upon heating above 350 K at 2.3 GPa. With temperature increasing one can also notice the continuous decrease in intensity of the connectivity band, signalling a lower level of hydrogen bonds.

## 6. Conclusions

The new experimental setups developed at the AILES beamline of Synchrotron SOLEIL considerably broaden the scope of infrared and THz spectroscopy on submillimetric samples. By combining the high brilliance and stability of synchrotron radiation with versatile optical configurations, precise control of temperature and pressure, and a comprehensive set of sample environments—including liquid cells, diamond anvil cells and uniaxial strain devices—these systems enable detailed investigations of both solid and liquid samples under extreme and well defined conditions. Their performance has been demonstrated through far-infrared measurements of water and ice across various thermodynamic states, revealing their capacity to probe subtle structural changes, hydrogen-bond dynamics and phase transitions.

These advancements extend beyond fundamental studies of simple molecular systems and thin-film materials, offering promising avenues for a broad spectrum of applications. The developed setups provide a sophisticated platform for *in situ* investigations, including the capability to perform measurements under applied electric or magnetic fields. This opens new opportunities to explore functional materials and emerging physical phenomena at the microscale.
